# Effects of training oncology physicians advising patients on complementary and integrative therapies on patient‐reported outcomes: 2‐year follow‐up of the multi‐center, cluster‐randomized KOKON‐KTO study

**DOI:** 10.1002/cam4.70008

**Published:** 2024-07-19

**Authors:** Alizé A. Rogge, Stefanie M. Helmer, Katja Icke, Claudia M. Witt

**Affiliations:** ^1^ Department of Psychosomatic Medicine, Center for Patient‐Centered Outcomes Research Charité–Universitätsmedizin Berlin Berlin Germany; ^2^ Institute of Health and Nursing Science Charité–Universitätsmedizin Berlin Berlin Germany; ^3^ Institute of Public Health and Nursing Research University of Bremen Bremen Germany; ^4^ Institute for Social Medicine, Epidemiology and Health Economics Charité–Universitätsmedizin Berlin Berlin Germany; ^5^ Institute for Complementary and Integrative Medicine University Zurich and University Hospital Zurich Zurich Switzerland

**Keywords:** cancer, follow‐up studies, integrative oncology, physician‐patient communication

## Abstract

**Purpose:**

Many cancer patients wish for complementary and integrative medicine (CIM) consultations led by their oncology physician. Within the KOKON‐KTO study, oncology physicians in the intervention group were trained in a blended learning to provide CIM consultations to their cancer patients in addition to distributing a leaflet about CIM websites. Control oncology physicians only distributed the leaflet. The training showed positive effects on the patient‐level. As of now, no consistent evidence exists on the long‐term effects of such one‐time‐only CIM consultation during cancer treatment.

**Methods:**

In the KOKON‐KTO follow‐up study, cancer patients previously participating in the KOKON‐KTO study (intervention group:IG and control group: CG) received, at least 24 months later, a follow‐up questionnaire by post, evaluating long‐term effects of the KOKON‐KTO consultation using the measures provided in the original study (patient‐physician communication (EORTC‐QLQ‐COMU2), satisfaction with cancer treatment (PS‐CaTE), CIM disclosure with healthcare provider (HCP), and need for CIM consultation during cancer therapy).

**Results:**

In total, 102 cancer patients participated in the follow‐up study (IG *n* = 62; CG *n* = 40). The overall reponse rate was around 36% (IG: 48.4%; CG: 23.7%). In the follow‐up study, differences between groups had increased and were still shown (EORTC‐QLQ‐COMU26, 0–100 point scale, ≥10‐point‐group difference) in some subscales: patient's active behavior (*in means;* IG:73.6 (95% CI, 63.8–83.5); CG:61.1 (95% CI, 52.4–69.8)); clinician‐patient relationship (IG:80.9 (95% CI, 71.8–90.0); CG:68.7 (95% CI, 59.3–78.0)). For some outcomes, differences decreased over time (e.g., EORTC‐QLQ‐COMU26 subscales “takes into account patient's preference” and “corrects misunderstandings”). More patients in the CG used CIM without oncology physicians' knowledge (IG: 13.7%, CG: 24.0%).

**Conclusion:**

This study presents first findings that one‐time‐only CIM consultations may enhance patient‐physican relationship and CIM disclosure long‐term. To further support cancer patients' in their wish for CIM consultations, training programs should provide oncology physicians with CIM competencies for different cancer stages including cancer survivors.

## 1 INTRODUCTION

Many studies suggest that complementary and integrative medicine (CIM)^1^ is used by a large number of cancer patients globally during their cancer treatment.^2^, ^3^ By now, clinical practice guidelines emphasize the use of CIM therapies to support symptom management, reduce anxiety or distress, and to enhance the patient‐physician communication by talking about CIM.^4^, ^5^ However, for many CIM therapies, there is conflicting, or no evidence at all to support such claims. This further complicates and restricts oncology physicians' communication about CIM in addition to a lack of CIM knowledge/education modules presented in medical study curricula.^6^, ^7^ To ensure the quality and evidence‐based advice of CIM consultations, cancer patients should be consulted by their oncology phycisian as there might be a risk of non‐adherence to cancer treatment if CIM consultations are conducted by non‐medical personnel.^8^

To close the gap between patients' CIM use and oncology physician's CIM communication skills, the Competence Network for Complementary Medicine (short KOKON)^9^ funded by the German Cancer Aid (grant number: Deutsche Krebshilfe; grants 109863 and 70112369) developed, amongst others, a decision‐making aid for cancer patients on finding reputable CIM providers,^10^ a CIM training framework for oncology physicians^11^ including the KOKON‐KTO consultation manual^12^, ^13^ and educational competencies for integrative oncology to be included in medical curricula.^14^, ^15^ Moreover, the subsequent KOKON‐KTO study evaluated the effects of a one‐time‐only CIM consultation led by oncology physicians on patient‐ and physician‐reported outcomes, compared to only providing a leaflet on reputable CIM websites alongside a brief introduction by the oncology physician. The study showed positive results, suggesting that the single intervention in the form of a structured CIM consultation has favorable effects on patient‐physician communication, patient satisfaction and readiness to make CIM decisions.^16^, ^17^

As of now, no consistent evidence exists on the long‐term effects of such one‐time CIM consultations in the course of cancer treatment.^18^, ^19^ It is unclear if patients seek out CIM support at all points of time during their cancer treatment, or only at specific time points; e.g. for symptom management during active cancer treatment or for disease processing at time of diagnosis. We previously reported on the effects of the KOKON‐KTO consultation on patient‐reported and physician‐reported outcomes during cancer treatment within the multicenter, cluster‐randomized KOKON‐KTO study.^16^, ^17^ The aim of this KOKON‐KTO follow‐up study was to report on the long‐term effects of the KOKON‐KTO consultation on patient‐reported outcomes.

## 2 METHODS

### 2.1 Study design

#### 2.1.1 KOKON‐KTO study

In the prospective, multicenter, cluster‐randomized KOKON‐KTO study,^16^, ^20^ we evaluated whether training oncology physicians to give advice on CIM to their patients with cancer, in addition to distributing an information leaflet about reputable CIM websites (intervention group), had different effects on outcomes at the patient, physician, and physician‐patient level compared to a one‐time short information consultation leaflet on reputable CIM websites (control group). Three websites providing information in German language were used (https://www.kokoninfo.de/komplementaermedizin, https://www.krebsinformationsdienst.de/behandlung/unkonv‐methoden‐index.php, https://www.onkopedia.com/de/my‐onkopedia/guidelines) and one international website in the English language (version used in the KOKON studies: https://www.mskcc.org/cancer‐care/diagnosis‐treatment/symptom‐management/integrative‐medicine/herbs; current version: https://www.mskcc.org/cancer‐care/diagnosis‐treatment/symptom‐management/integrative‐medicine/herbs ). Oncology physicians in both groups were asked to provide advice for up to 10 of their cancer patients (one consultation per patient for a maximum of 10 patients per oncology physician), and to complete standardized questionnaires.

The study was conducted from February 15, 2018 until November 30, 2018. In total, 297 patients (128 in the intervention group and 169 in the control group) received advice from 41 (*n* = 18 for the intervention group and *n* = 23 for the control group) randomized oncology physicians. At the end of the KOKON‐KTO study, oncology physicians were invited to participate in the KOKON‐KTO training as an incentive for participation in the study trial, and to conduct KOKON‐KTO consultations with up to five cancer patients on a voluntary basis. For more information, see publications on KOKON‐KTO study results.^16^, ^17^

#### 2.1.2 KOKON‐KTO follow‐up study

Starting from November 2020, cancer patients were asked to participate in the KOKON‐KTO follow‐up study to provide further information on the effects of the one‐time CIM consultation (KOKON‐KTO consultation + information consultation on reputable CIM websites) or a one‐time short information consultation on reputable CIM websites. Study information, follow‐up study consent, and questionnaires were sent to all KOKON‐KTO study participants via postal service. Questionnaires included validated as well as self‐formulated items used previously in the KOKON‐KTO study. Pre‐paid return envelopes were sent out with each study invitation to facilitate participation. A reminder was sent out in February 2021.

##### Eligibility criteria

Patients were eligible if they participated in the KOKON‐KTO study and lived in the German counties for which the ethical approval was received for the KOKON‐KTO follow‐up study (see ethics approval section). Moreover, participants had to be in good enough health to fill out questionnaires on their own without assistance.

### 2.2 Quantitative outcomes and outcome measures

Patients were asked to complete questions on quality of communication (EORTC‐QLQ‐COMU26^21^; scales and single items range from 0 to 100), satisfaction with cancer treatment (PS‐CaTE^22^; scale ranges from 1 to 5), current or planned CIM therapy and experience (self‐developed; bases on KOKON‐KTO study), and use of websites from the KOKON‐KTO flyer (self‐developed; based on KOKON‐KTO study^20^). Questionnaires were included due to their relevance in the original KOKON‐KTO study to allow for comparibility, as well as on the basis of a consensus procedure on outcomes and outcome measures.^23^

Additionally, patients completed self‐developed questions on changes in their cancer therapy since the KOKON‐KTO study participation, use of CIM without disclosure to healthcare provider (HCP), occurrence of additional CIM consultation, general necessity of CIM consultation in routine care, satisfaction with information on CIM, and motivation and interest on using CIM additional to cancer therapy.

### 2.3 Statistical analysis

The results will be shown as descriptive analyses. All questionnaires will be scored and calculated according to their respective manuals. Continuous variables are presented as means and standard deviations, and categorical variables are presented as absolute and relative frequencies. As a guide to determine group differences, we applied a cut‐off of ≥ 10 points to indicate moderate differences. The threshold was chosen by the author team as no guideline on how to define meaningful differences in groups exists for the measures used. Analyses are considered exploratory, and were conducted with IBM SPSS Statistics, version 27.0.^24^

Baseline characteristics were extracted from the main KOKON‐KTO study data.^16^

### 2.4 Qualitative data collection and analysis

In open‐ended questions, participants were asked to indicate all CIM therapies they used, but not already mentioned in the questionnaires. Additionally, they could disclose further information on changes in their cancer therapy since study participation, CIM therapies in use in the case of non‐disclosure to healthcare provider (HCP), reasons for additional CIM consultations (if provided) as well as a desire for additional CIM consultations and reasons for their participation in the KOKON‐KTO study.

Qualitative content analysis was performed according to Mayring^25^ using MAXQDA,^26^ a qualitative data analysis software. The research team predefined deductive codes according to the questionnaire items presented, other subcategories were created in a continuous process of inductively building codes. All results were discussed amongst the author team.

## 3 RESULTS

The KOKON‐KTO follow‐up study was conducted between November 2020 and April 2021 (last questionnaire returned). Characteristics of oncology physicians and cancer patients participating in the initial trial are presented elsewhere.^16^, ^17^ In total, 102 cancer patients participated in the KOKON‐KTO follow‐up study (intervention group *n* = 62; control group *n* = 40). The overall response rate was 36%, with more patients participating from the intervention group (48.4%) than from the control group (23.7%). For some participants (*n* = 8), reasons for non‐participation were disclosed to the study team (intervention group: death (*n* = 1), no interest (*n* = 2), due to health reasons not able (*n* = 3); control group: death (*n* = 1), due to health reasons not able (*n* = 1)).

Mostly, female cancer patients participated in the KOKON‐KTO follow‐up study (females in KOKON‐KTO study: intervention group (*n* = 110; 85.9%) and control group (*n* = 140; 82.8%); females in KOKON‐KTO‐Follow‐up: intervention group (*n* = 49; 94.2%) and control group (*n* = 44; 88.0%)). Follow‐up participants age was comparable between both evaluation time points (KOKON‐KTO study: intervention group (mean 54.1 SD ±10.6) and control group (mean 55.6 SD ±11.8); follow‐up: intervention group (mean 54.1 SD ±10.2) and control group (55.9 SD ±12.0)).

### 3.1 Quantitative outcomes

Participants from the former intervention group rated their “active role behavior,” “clinician‐patient‐relationship,” and the degree of privacy higher (≥10 points) than patients from the former control group using the EORTC‐QLQ‐COMU26 questionnaire (see Table 1). Moreover, in comparison to the results from the KOKON‐KTO study, these scales were rated even higher in the follow‐up than in the baseline study. Even though other scale results decreased in their ratings over time, the intervention group always indicated higher communication skills in their consultations with their oncology physicians than the control group (see Table 1).

**TABLE 1:** Quantitative Results from the KOKON KTO study and the KOKON KTO Follow‐Up study.

	KOKON KTO study			KOKON KTO Follow‐up		
	Intervention group (*n* = 122) mean (95% CI)	Control group (*n* = 154) mean (95% CI)	Point difference (Intervention vs. Control)	Intervention group (*n* = 32) mean (95% CI)	Control group (*n* = 28) mean (95% CI)	Point difference (Intervention vs. Control)
EORTC‐QLQ‐COMU26^1,^ ^a^						
Patient's active role behaviors	85.0 (80.0–90.0)	74.3 (70.0–78.7)	10.7	73.6 (63.8–83.5)	61.1 (52.4–69.8)	12.5
Clinician‐patient relationship	87.2 (82.1–92.4)	76.1 (71.5–80.7)	11.1	80.9 (71.8–90.0)	68.7 (59.3–78.0)	12.2
Professional's qualities to create a relationship	89.6 (85.1–94.1)	80.8 (76.8–84.8)	8.8	83.5 (75.4–91.7)	73.6 (64.8–82.4)	9.9
Professional's skills	90.3 (86.4–94.1)	84.5 (81.0–87.9)	5.8	85.2 (77.8–92.5)	80.4 (73.2–87.5)	4.8
Professional's management of patient's emotions	85.6 (80.7–90.5)	76.9 (72.5–81.2)	8.7	76.4 (66.7–86.1)	70.6 (60.8–80.5)	5.8
Professional takes into account patient's preference	78.8 (71.8–85.9)	67.3 (61.1–73.5)	11.5	68.8 (58.2–79.3)	63.1 (52.3–73.8)	5.7
Professional corrects misunderstandings	84.8 (79.6–90.0)	71.5 (66.9–76.1)	13.3	69.8 (57.8–81.8)	66.7 (56.7–76.6)	3.1
Professional's skills related to information	73.6 (67.4–79.8)	58.3 (52.8–63.8)	15.3	60.4 (49.3–71.5)	56.3 (47.0–65.7)	4.1
Enough privacy	85.0 (79.4–90.6)	74.0 (69.1–78.9)	11.0	82.3 (72.6–91.9)	70.2 (58.4–82.1)	12.1

	Intervention group mean (95% CI)/*N* (%)	Control group mean (95% CI)/*N* (%)	Point difference (Intervention vs. Control)	Intervention group mean (95% CI)/*N* (%)	Control group mean (95% CI)/*N* (%)	Point difference (Intervention vs. Control)
PS‐CaTE^2,^ ^b^ (mean)	4.2 (4.0–4.4)	3.7 (3.5–3.8)	0.5	4.0 (3.7–4.2)	3.5 (3.2–3.7)	0.5

*Note*: 1, KOKON‐KTO study; 2, KOKON‐KTO follow‐up.

^a^ EORTC‐QLQ‐COMU: higher scores indicate better communication (0–100).

^b^ PS‐CaTe: higher values indicate higher satisfaction (1–5); for all results, column two indicated timepoint of data retrival.

Both groups indicated, in the follow‐up evaluation, satisfaction with their cancer therapy (PS‐CaTE: intervention group 4.0 [95% CI, 3.7–4.2]; control group 3.5 [95% CI, 3.2–3.7]). The majority of participants found CIM consultation to be a necessary part in cancer therapy (intervention group: 76.0%; control group: 70.0%); however, more participants in the intervention group were interested in CIM use (quite a lot or very much: 63.4%) than in the control group (quite a lot or very much: 40.8%) (see Table 2).

**TABLE 2:** Descriptive results of cancer patients participating in the KOKON‐KTO Follow‐up by intervention/control group.

	Intervention group *n* (%)	Control group *n* (%)
Satisfaction with CIM Consultation (yes)	30 (57.7%)	19 (66.0%)
Necessity of CIM consultation in routine care (quite a lot–very much)	38 (76.0%)	35 (70.0%)
Motivation for CIM use (quite a lot & very much)	27 (53.0%)	22 (44.0%)
Interest in CIM use (quite a lot & very much)	33 (63.4%)	20 (40.8%)
Use of websites from the KOKON‐KTO flyer^a^ (yes)		
Kokoninfo.de	33 (25.8%)	30 (17.8%)
Krebsinformationsdienst.de	30 (23.4%)	39 (23.1%)
Memorial Sloan Kettering	5 (3.9%)	4 (2.4%)
Onkopedia.com	15 (11.7%)	13 (7.7%)
None	2 (1.6%)	50 (29.6%)
Frequency of usage	0–1: 25 (19.5%) 1–4: 22 (17.2%) ≥5: 3 (2.3%)	0–1: 25 (14.8%) 1–4: 23 (13.6%) ≥5: 2 (1.2%)

^a^ Higher scores indicate higher ratings on the usefulness of websites (0–10).

Overall, cancer patients from both groups mainly used the websites Kokoninfo.de (intervention group: 25.8%; control group: 17.8%) and krebsinformationsdienst.de (intervention group: 23.4%; control group: 23.1%) as information websites listed in the KOKON‐KTO CIM information leaflet (see Table 2).

About one‐fifth of patients from each group asked for further CIM consultations (intervention group: 20.0%; control group: 18.0%), and more participants from the control group disclosed current CIM use without HCPs knowledge (intervention group: 13.7%; control group: 24.0%) (see Table 3).

**TABLE 3:** Results on CIM use in the KOKON‐KTO Follow‐up study.

	Intervention group (*n* = 62) *n* (%)	Control group (*n* = 40) *n* (%)
Changes in cancer therapy		
Yes	11 (21.6%)	8 (4.7%)
Current CIM treatments without disclosure to HCP		
Yes	7 (13.7%)	12 (24%)
Asked for further CIM consultation		
Yes	10 (20%)	9 (18%)
*Rating of current CIM therapy use*		
Acupuncture/Acupressure		
Positive	1 (0.8%)	3 (1.8%)
Unclear		1 (0.6%)
Anthroposophical medicine		
Positive	1 (0.8%)	1 (0.6%)
Unclear	2 (1.6%)	
Chinese herbs		
Positive	1 (0.8%)	1 (0.6%)
Chiropractic		
Positive	0	2 (1.2%)
Homeopathy		
Positive	14 (10.9%)	12 (7.1%)
Unclear	1 (0.8%)	0
Negative	2 (1.6%)	0
Neural therapy		
Positive	1 (0.8%)	0
Osteopathy		
Positive	8 (6.3%)	8 (4.7%)
Unclear	1 (0.8%)	0
Misteltoe		
Positive	1 (0.8%)	2 (1.2)
Unclear	0	1 (0.6%)
Other phytotherapy		
Positive	6 (4.7%)	1 (0.6%)
Unclear	1 (0.8%)	2 (1.2%)
Qigong/Tai Chi		
Positive	5 (3.9%)	3 (1.8%)
Yoga		
Positive	12 (9.4%)	7 (4.1%)
Unclear	0	1 (0.6%)
Relaxation exercises		
Positive	14 (11%)	13 (7.7%)
Unclear	0	1 (0.6%)
Others		
Positive	14 (10.9%)	8 (4.7%)
Unclear	1 (0.8%)	1 (0.6%)

Abbreviations: CIM, complementary and integrative medicine; HCP, healthcare provider.

### 3.2 Qualitative outcomes

#### 3.2.1 Control group

Participants reported use of additional CIM treatments that were not mentioned in the questionnaires. These patient‐reported CIM treatments presented a broad definition of CIM from nutrition supplements (e.g., “high‐dosage vitamin c”) to informational books about “anti‐cancer nutrition” as well as psychotherapy or “spirituality” (*n* = 12). Reasons for participation in the KOKON‐KTO study can be categorized in two sections: “Interest in CIM” (seeking for physician recommendations; symptom management; survival) and “Patient Engagement” (e.g., supporting patient‐centered outcome research). Patients felt generally well‐informed about CIM, and wanted their oncology physicians to be equipped with detailed CIM recommendations. Participating in the KOKON‐KTO study made participants of the control group feel “empowered knowing to be able to talk to oncology physicians about CIM interest.”

Patients asked for additional CIM consultations when there was a change in their treating oncology physician, when they wanted information on finding reputable CIM providers, or when they wanted CIM updates for symptom management during cancer therapy. Some patients from the control group reported to feel “not entirely informed about CIM and wished to receive further information on treatments.” Additionally, participants highlighted to seek “tailored CIM information for their current status of cancer treatment,” especially for the cancer survivor stage. Some patients also indicated to lack overall information on their cancer treatment (e.g., “why am I in 5‐year cancer aftercare despite complete remission?”).

#### 3.2.2 Intervention group

Participants reported to use additional treatments such as nutrion supplements, as well as homeopathy (*n* = 7). As reasons for participation in the KOKON‐KTO study, participants stated three main reasons as in (1) “interest in CIM” (“hope” in CIM treatment, CIM use as cancer survivor; general information, symptom management); (2) “patient engagement” (support of CIM research); (3) “physician's recommendation to participate.” Participants from the intervention group stated to have asked for and received further CIM consultations pertaining to: symptom management (*n* = 5), further information on specific CIM treatment (*n* = 3) and medication interactions of nutrition supplements (*n* = 2). During the KOKON‐KTO consultations, participants stated to lack advice on reputable CIM providers and CIM recommendations for cancer survivors. Moreover, some patients indicated that an absence of follow‐up consultations left participants feeling “alone with CIM” after the initial conversation. Participants postiviely mentioned that oncology physicians asked for more time to research on specific CIM treatment of interest, which made them feel “heard and taken seriously.” Overall, they felt to have received more time in consultations following the KOKON‐KTO consultation with their oncology physician.

## 4 DISCUSSION

The KOKON‐KTO follow‐up study presented the first findings on long‐term effects of a one‐time‐only CIM consultation for cancer patients provided by their oncology physician. Even 2 years after the initial CIM consultation, the KOKON‐KTO consultation still showed positive effects on many patient‐reported outcomes such as better patient‐physcian‐relationship.

In addition, the results of the follow‐up study showed that patients were still interested in CIM, and tended to use CIM without HCP disclosure more often if no strucutured CIM consultation was presented before. In the case of a CIM consultation, patients felt more active in their role behavior and stated their patient‐physician relationship was more effective than without the CIM consultation. Roydhouse et al.^27^ described similar findings using data from the large population‐based Cancer Care Outcomes Research and Surveillance (CanCORS) study, in which lung and colorectal cancer patients reported higher medical care experience and care quality when disclosing phytotherapy use to HCP than without CIM disclosure. However, as the 10‐point threshold was chosen by the author team to facilitate the discussion of results, no further evidence exists to this threshold actually showing a relevant clinical difference.

Due to the type of longitudinal data assessment, participants from the KOKON‐KTO study might have gone through changes in their cancer stage. As such, participants wished for tailored CIM advice for their current cancer therapy which the KOKON‐KTO consultation training^13^ did not prepare participating oncology physicians for. As an interesting approach to different CIM advice settings within varied cancer stages, Weeks et al.^28^ recommend a 3‐phase model for early stage (initial diagnosis phase), mid stage (maintenance phase) and late stage (survivorship or palliative care phase) with CIM advice adapting to cancer trajectory, therapy aims and patterns of information‐seeking.

Some studies refer to unsupervised CIM use as potential risk for the overall cancer therapy outcome.^8^ In the KOKON‐KTO follow‐up study, no negative events due to CIM use were reported, despite the presence or absence of a structured CIM consultation. However, all patients in this study received at least basic information on where to find reputable CIM online information, and were included in an evidence‐based medical oncology treatment. For the KOKON‐KTO setting of CIM use during cancer therapy, other studies reported well‐informed cancer patients as reasons for unharmful CIM use as well.^29^

The KOKON‐KTO study was conducted in two phases. First, the intervention group was trained to conduct the KOKON‐KTO consultation with their patients. Only in the second phase, and as an incentive for participating oncology physicians from the control group, the control group received the opportunity to participate in the KOKON‐KTO training and to give consultations to their cancer patients. Due to this and delays in the acceptance of the amendment by the resident ethics committees, the follow‐up study was conducted with cancer patients 2 years after the KOKON‐KTO study. Consequently, due to delayed training of control group physicians, some participants of the control group might have additionally received the KOKON‐KTO study intervention after their physicians were trained in the KOKON‐KTO consultation which might have had an effect on the results presented. Moreover, as KOKON‐KTO study participants were not limited to CIM use within treatment setting, participants might have received additional CIM advice elsewhere which might influence the follow‐up study results. This is a limitation, that might have resulted in lower group differences. Furthermore, the patients received the consultation only once, which may have limited impact. However, most of the cancer patients visit their oncology physicians subsequently during cancer therapy and we cannot rule out that follow‐up conversations may have occurred.

About one‐third of cancer patients from the KOKON‐KTO study participated in the follow‐up study. In general, reasons of non‐participation of the other two‐third of KOKON‐KTO participants remained unclear. To our knowledge, no long‐term data exists to compare response‐rates for a single CIM consultation intervention. As cancer patients were not initially informed about the follow‐up study, dropout rates might be explained by the lack of commitment when asking again for an additional informed consent. Considering adherence rates from another CIM intervention study which recommended cancer patients, after their first CIM consultation, to attend to a follow‐up consultation, reported a response rate of 26%. Reasons for the low adherence rate were discussed in the frame of CIM follow‐ups not being relevant for all patients, but rather for those more likely to access additional clinical services.^30^ Another factor for the KOKON‐KTO follow‐up adherence rate might be that no strategy for further dissemination of KOKON‐KTO materials, such as criteria list on how to seek for reputable CIM providers, was actively promoted beyond the KOKON‐KTO study in the participating sites. Subsequently, this may be a further reason for lost to follow‐up participation.^31^

We were not able to rule out possible cross‐contamination between oncology physicians in the study. However, oncology physicians included in the study worked in hospitals or private practices across Germany which reduced the chance of exchange of information between participants.

In our study we received additional information on patients subsequent use of CIM therapies, changes in cancer care as well as reasons for behavior and changed cancer therapies. For this, we only used open‐ended questions in our questionnaire that do not take up too much of the patient's time. However, this may have resulted in underreporting. Future research could take up the subject in in‐depth qualitative interviews.

## 5 CONCLUSION

This is the first study following cancer patients after a one‐time‐only CIM consultation intervention (KOKON‐KTO consultation) 2 years later. Conducting structured, evidence‐based CIM consultations by the oncology physician with their cancer patients still seems to show beneficial effects on patient‐physican relationship, patient's active role behavior as well as a higher sense of privacy within the consultation. Both groups still indicated that they wished for further CIM information in the course of illness, especially at the cancer survivor stage. The KOKON‐KTO study indicated that structured, evidence‐based CIM consultation, as an integrative part of cancer therapy, show no harmful effects on CIM use, and leave patients feeling informed, considered, and seen in their needs during cancer therapy.

## AUTHOR CONTRIBUTIONS

**Alizé A. Rogge:** Conceptualization (equal); data curation (equal); formal analysis (equal); investigation (equal); methodology (equal); project administration (equal); writing – original draft (lead). **Stefanie M. Helmer:** Project administration (equal); writing – review and editing (equal). **Katja Icke:** Data curation (lead); project administration (supporting); writing – review and editing (equal). **Claudia M. Witt:** Conceptualization (equal); funding acquisition (lead); investigation (equal); methodology (equal); writing – original draft (equal).

## FUNDING INFORMATION

This project was part of the Competence Network for Complementary Medicine in Oncology, which was established as a collaborative research project funded by German Cancer Aid (Deutsche Krebshilfe; grants 109863 and 70112369) and the Günter und Regine Kelm Stiftung. The funder of the study played no role in the study design, data collection, data analysis, data interpretation, or writing of the report. The corresponding author had full access to all of the data in the study and had final responsibility for the decision to submit the manuscript for publication.

## CONFLICT OF INTEREST STATEMENT

Alizé A. Rogge reports reimbursements for travel (full or partial). Claudia M. Witt reports fees for scientific presentations from Swiss hospitals; and board roles in the Society of Acupuncture Research, the Society of Integrative Oncology, Schweizer Fachverband für Mind Body Medicine. The other authors made no disclosures.

## ETHICS STATEMENT

Respective ethics committees approved the amendment for the KOKON‐KTO follow‐up study (ethics committee of Charité–Universitätsmedizin Berlin [EA1/127/17], Baden‐Wuerttemberg Medical Association [B‐F‐2017‐10], Nord Rhine Medical Association [2417337], ethics committee of the Medical Association of Westphalia‐Lippe [2017‐624‐b‐S], ethics committee of the Medical Faculty of Würzburg [274/17_z‐me], ethics committee of the Medical Faculty of Heidelberg [S‐550/2017], and ethics commission of the Albert Ludwigs University of Freiburg [531/17]). One ethics committee was not contacted to permit the amendment for the KOKON‐KTO follow‐up study as less than 5 cancer patients participated from that German county and to reduce administration efforts and expenses (Hamburg Medical Association [MC‐368/17]). Informed consent was obtained from all of the participants.

## CLINICAL TRIAL REGISTRATION

The trial registration number of the KOKON‐KTO study is DRKS00012704 on the German Clinical Trials Register (registered August 28, 2017).

## Data Availability

The data sets analyzed during the current study are available from the corresponding author on reasonable request.
